# Language dysfunction correlates with cognitive impairments in older adults without dementia mediated by amyloid pathology

**DOI:** 10.3389/fneur.2023.1051382

**Published:** 2023-05-17

**Authors:** Chunchen Xiang, Weiping Ai, Yumei Zhang, Alzheimer's Disease Neuroimaging Initiative

**Affiliations:** ^1^Department of Neurology, Beijing Tiantan Hospital, Capital Medical University, Beijing, China; ^2^Department of Neurology, Zhangjiakou First Hospital, Zhangjiakou, China; ^3^Department of Rehabilitation Medicine, Beijing Tiantan Hospital, Capital Medical University, Beijing, China; ^4^Center of Stroke, Beijing Institute for Brain Disorders, Beijing Key Laboratory of Translational Medicine for Cerebrovascular Disease, Beijing, China; ^5^China National Clinical Research Center for Neurological Diseases, Beijing, China

**Keywords:** Alzheimer's disease, confrontation naming, semantic fluency, amyloid, tau, neurodegeneration

## Abstract

**Background:**

Previous studies have explored the application of non-invasive biomarkers of language dysfunction for the early detection of Alzheimer's disease (AD). However, language dysfunction over time may be quite heterogeneous within different diagnostic groups.

**Method:**

Patient demographics and clinical data were retrieved from the Alzheimer's Disease Neuroimaging Initiative (ADNI) database for the participants without dementia who had measures of cerebrospinal fluid (CSF) biomarkers and language dysfunction. We analyzed the effect of longitudinal neuropathological and clinical correlates in the pathological process of semantic fluency and confrontation naming. The mediation effects of AD biomarkers were also explored by the mediation analysis.

**Result:**

There were 272 subjects without dementia included in this analysis. Higher rates of decline in semantic fluency and confrontation naming were associated with a higher risk of progression to MCI or AD, and a greater decline in cognitive abilities. Moreover, the rate of change in semantic fluency was significantly associated with Aβ deposition, while confrontation naming was significantly associated with both amyloidosis and tau burden. Mediation analyses revealed that both confrontation naming and semantic fluency were partially mediated by the Aβ aggregation.

**Conclusion:**

In conclusion, the changes in language dysfunction may partly stem from the Aβ deposition, while confrontation naming can also partly originate from the increase in tau burden. Therefore, this study sheds light on how language dysfunction is partly constitutive of mild cognitive impairment and dementia and therefore is an important clinical predictor.

## Introduction

With the global increase in the aged population, the socio-economic burden of Alzheimer's disease (AD) has been continuously up-ticking on both patients and their caregivers (1, 2). The pharmacological treatments show a poor risk–benefit relationship because of the frequent discontinuation and limited improvement in patients' health conditions (3, 4). Recent studies suggest that the reduction of etiological risk factors and multidomain interventions could prevent cognitive decline and dementia in AD patients (5). Importantly, missing disease symptoms and a delayed diagnosis can significantly disturb the treatment initiation, especially in the early stage, resulting in a poor outcome (6). Amyloid-β (Aβ) and tau aggregation-associated neurodegeneration might start decades before the actual manifestation of AD symptoms, whose presence could be recognized as the AD continuum according to the biological definition of AD based on the ATN (Aβ deposition [A], pathologic tau [T], and neurodegeneration [N]) hallmarks (7, 8). Amyloid deposition might accelerate tau pathology and then lead to neurodegeneration (9), but certain inconveniences of these biomarkers in practical scenarios prevent their further clinical usage.

There has been a consensus about language dysfunction in AD and related dementia cases, while the disease-related language features and their implications in mild cognitive impairment (MCI) diagnosis are not well-characterized (10). Previous studies have found that semantic fluency and name recalling are initially affected by the breakdown of other facets in these patients (11). Semantic fluency has been found to segregate MCI subjects from healthy controls and can be exploited to predict the risk of pathological conversion of MCI to AD (12, 13). Naming tasks permit clinicians to gauge the extent of lexical–semantic disintegration and confrontation with naming, thereby indicating that the disorders are not simply resulting from the age-associated decline in semantic and/or visuoperceptual knowledge (14, 15). MCI patients have been found to retrieve names of famous persons or public figures, even after the deliverance of a semantic cue, which is shown as the major complaint among MCI and AD patients (16).

Although language dysfunction is common in the early stage of typical AD, the mechanism underlying the pathological process is still unexplored. Recently, it has been revealed that Aβ accumulation may have a significant impact on the language network in the early stage of AD (17). Another study shows that an increased burden of pathological tau aggregates may also associate with declined capacity in name memorization in the primary progressive aphasia with underlying AD neuropathology (18). However, the implications of amyloid and tau pathologies underlying the AD pathomechanism are not well-defined. Therefore, we evaluated the longitudinal associations of the progression of language impairment in animal confrontation naming in both cognitively unimpaired (CU) and MCI participants based on the expression of neurocognitive and neuroimaging markers of AD. Furthermore, we explored the potential mechanisms of language impairment and whether the ATN framework might mediate the progression of AD pathology.

## Methods

### Study population

Clinical and pathological data of the participants were retrieved from the Alzheimer's Disease Neuroimaging Initiative (ADNI) database (https://adni.loni.usc.edu/), including the expression of cerebrospinal fluid (CSF) biomarkers, positron emission tomography (PET), and neuropsychological assessment. As a public–private partnership, the ADNI database is led by the principal investigator, Michael W. Weiner, MD, and participants between 55 and 90 years of age who are regularly recruited at 57 sites in the United States and Canada. The participants undergo a series of initial tests that are repeated at intervals over subsequent years. For up-to-date information on AD and MCI, see www.adni-info.org. This study included 272 ADNI participants who completed their neuropsychological assessments and CSF biomarker screening tests with a follow-up for 3 years at least. Definitions of the participant classifications, including CU and MCI, have been described elsewhere (19). The threshold value of CSF Aβ1-42 was 880 pg/ml or 1.11 SUVR for the FBP PET scan. CSF pTau was used to assess T+ with a cutoff value of more than 26.64 pg/mL or 1.37 SUVR for the FTP PET scan, while N+ was considered when tTau ≥300 pg/mL (19).

### Neuropsychological assessment

The 60-item version of the Boston naming test (BNT) and category semantic fluency animals were used for the assessment of confrontation naming and semantic fluency. The lower score indicates an elevated level of language dysfunction. The optimal cutoff value for semantic fluency and confrontation naming at baseline were 13 (20) and 22 (21), respectively.

For the evaluation of global and specific cognition domains, we used the Mini-Mental State Examination (MMSE) and AD Assessment Schedule-Cognition (ADAS-Cog), respectively. The immediate recall, learning scope, delayed recall, and recognition from the Rey auditory learning test were used for the assessment of immediate memory, while the Trail Making Test parts A and B were employed for the assessment of processing speed and executive functions. The Digit Span Backward and Digit Span Forward tests were applied to assess the attention and working memory, while the command and copy tasks of the Clock Drawing Test were used for the visuospatial functional analysis. Functional activities were evaluated by the functional activity questionnaire, and neuropsychiatric symptoms were identified using the Geriatric Depression Scale and Neuropsychiatric Inventory Questionnaire. The higher score on several scales indicates an elevated level of specific cognitive domains, including global cognition, processing speed, execution, functional activities, and neuropsychiatric symptom. Supplementary material 1 was available for detailed information about the neuropsychological assessment.

### Clinical disease progression

Patients were assigned to the clinical disease progression group if their clinical manifestations changed over time compared to others who had stable disease conditions (hereafter, the stable group). Additionally, the participants who did not meet the criteria of AD and/or MCI at follow-up were also assigned to the stable group (22).

### Statistical analyses

A *t*-test was used to compare the demographic and clinical data between the CU and MCI groups. One-way analysis of variance (ANOVA) with Tukey's correction was used for the continuous variables, while the chi-squared (χ2) test was applied for the categorical variables to compare the demographic and clinical data across different ATN profiles at the baseline and follow-up. The correlation of study variables (the baseline and mean change rate of semantic fluency and confrontation naming) with demographic and clinical characteristics at the baseline were assessed by the Pearson correlation test for continuous variables and the chi-squared (χ2) test for categorical variables, while the outliers with mean more than three times of SD were also excluded.

The linear model was used to determine the association of global cognition and executive function with the baseline and change rate of semantic fluency and confrontation naming controlling for age, sex, education, and APOE4 status. We use linear models to further explore the independent influence of Aβ and tau pathologies on the baseline and change rate of semantic fluency and confrontation naming by including CSF Aβ and tau and the predictors in the multivariable model, controlling for the same covariables above. Furthermore, the Kaplan–Meier survival curve and the Cox proportional hazards model were used to evaluate the predictive abilities of changes in language assessments for the disease progression (to MCI or AD). Survival curves of clinical progression were plotted based on the mean annual change in semantic fluency and confrontation naming, while the median of change rate was used as cutoff values.

**Figure 1:**
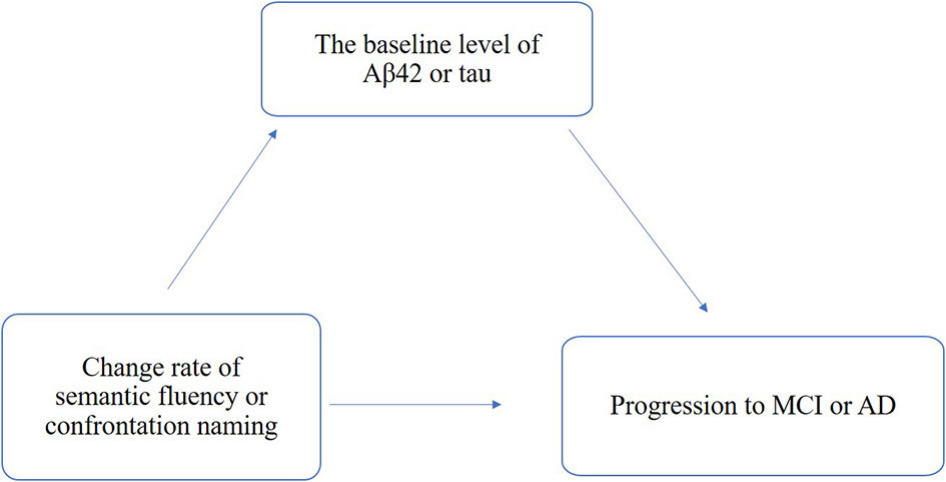
Schematic model of amyloid and tau deposition as the mediator between the change rate of semantic fluency and confrontation naming and clinical progression.

Moreover, we performed mediation analyses with bootstrapping (5,000 iterations) methods to analyze the mediation effects of Aβ and tau/phospho-tau accumulations on the associations of change rates in confrontation naming and semantic fluency for the cognitive decline and clinical progression, while we adjusted age, years of education, sex, and APOE4 status based on the schematic model as shown in Figure 1. All statistical analyses were performed using R version 4.1.2 with the package “glm,” “ggplot2,” “mediation,” “survival,” and “arm” and SPSS 23.0. All tests used a significance level of a *p-*value of < 0.05.

## Results

### Demographic characteristics

A total of 272 non-demented elderly individuals with the baseline and follow-up data of neuropsychological and CSF biomarker assessments were included in this study. The age of participants ranged from 54 to 89 years and years of education from 12 to 20 years. The study population had 36.5% of female participants and 43% of APOE4-positive subjects.

Semantic fluency and confrontation naming at the baseline were positively correlated with the decline of global cognition and specific cognitive domains, including executive function (Supplementary Tables 1, 2). Lower semantic fluency was associated with poorer global cognition (β = −0.326, *p* < 0.001) and executive function (β = −0.342, *p* < 0.001). Poorer confrontation naming was also assessed with the decline in global cognition (β = −0.361, *p* < 0.001) and executive function (β = −0.466, *p* < 0.001) (Supplementary Table 3).

### Longitudinal analysis

**Figure 2:**
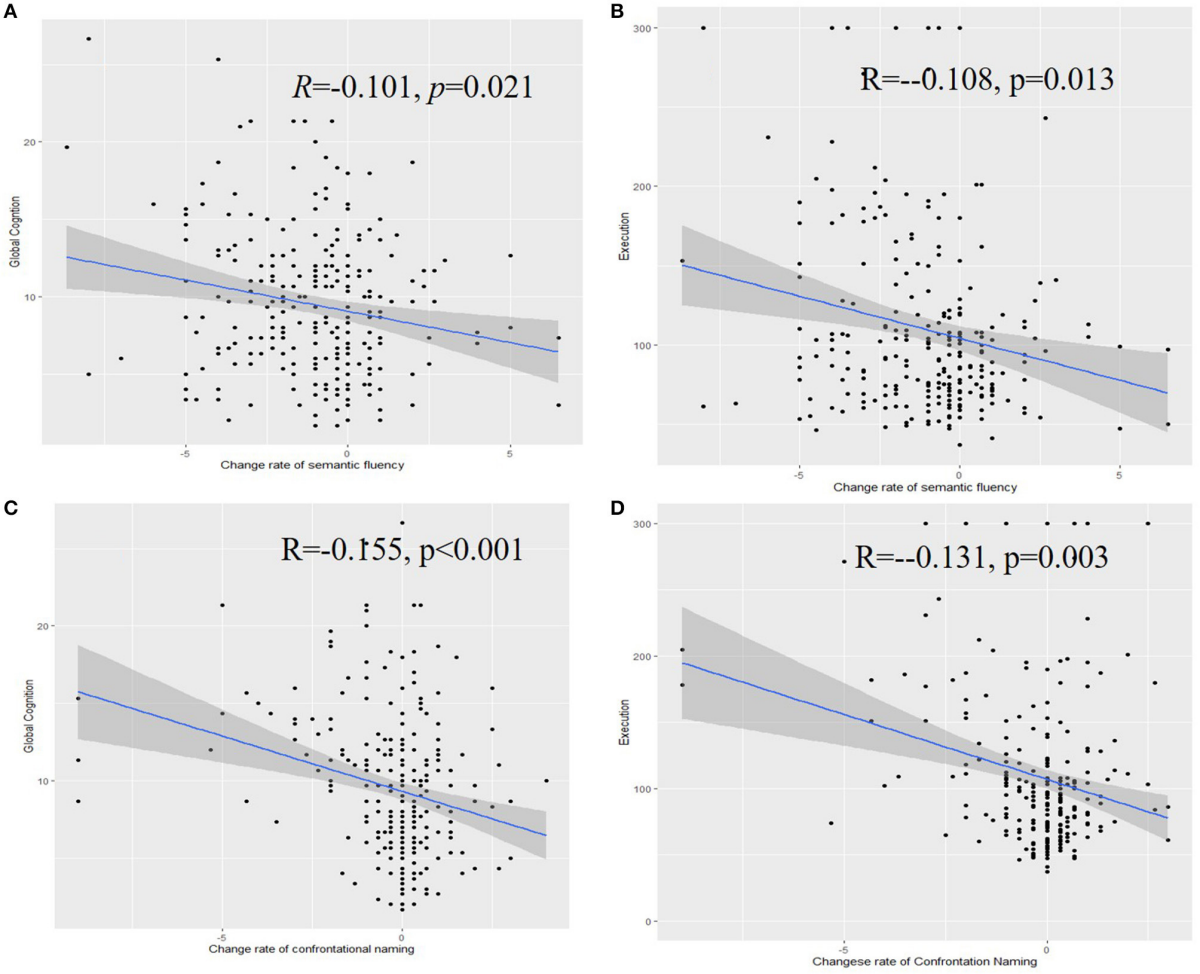
Associations of the rate of change of semantic fluency with the global cognition **(A)**, executive function **(B)**, confrontation naming with the global cognition **(C)**, and executive function **(D)**.

The yearly differences (standard deviation; SD) in confrontation naming and semantic fluency test results were, respectively, 0.26 (1.48) and 1.06 (2.23). A higher rate of decrease in confrontation naming was associated with poorer cognition (*R* = −0.155, *p* < 0.001), executive function (*R* = −0.131, *p* = 0.003), and functional activity (*R* = −0.188, *p* < 0.001) at the baseline. Moreover, a higher rate of decrease in confrontation naming was also related to immediate recall (*R* = 0.159, *p* < 0.001), learning ability (*R* = 0.109, *p* = 0.017), delayed recall (*R* = 0.182, *p* < 0.001), and delayed recognition (*R* = 0.107, *p* = 0.019) (Figure 2).

A higher rate of decrease in semantic fluency was associated with poorer cognition (*R* = −0.101, *p* = 0.021), executive function (*R* = −0.108, *p* = 0.013), and functional activity (*R* = −0.135, *p* = 0.005) at the baseline. Similarly, a higher rate of decrease in confrontation naming was associated with immediate recall (*R* = 0.140, *p* = 0.001), learning ability (*R* = 0.141, *p* = 0.002), delayed recall (*R* = 0.140, *p* = 0.002), and delayed recognition (*R* = 0.108, *p* = 0.018).

The relationship between the change rate of semantic fluency and confrontation naming and global cognition is as follows. The greater decline of semantic fluency was associated with poorer global cognition (β = −0.326203, *p* = 0.001) and executive function (β = −0.302, *p* < 0.001). Poorer confrontation naming was also assessed with the decline in global cognition (β = −0.174, *p* = 0.003) and executive function (β = −0.213, *p* < 0.001) (Supplementary Table 4).

**Figure 3:**
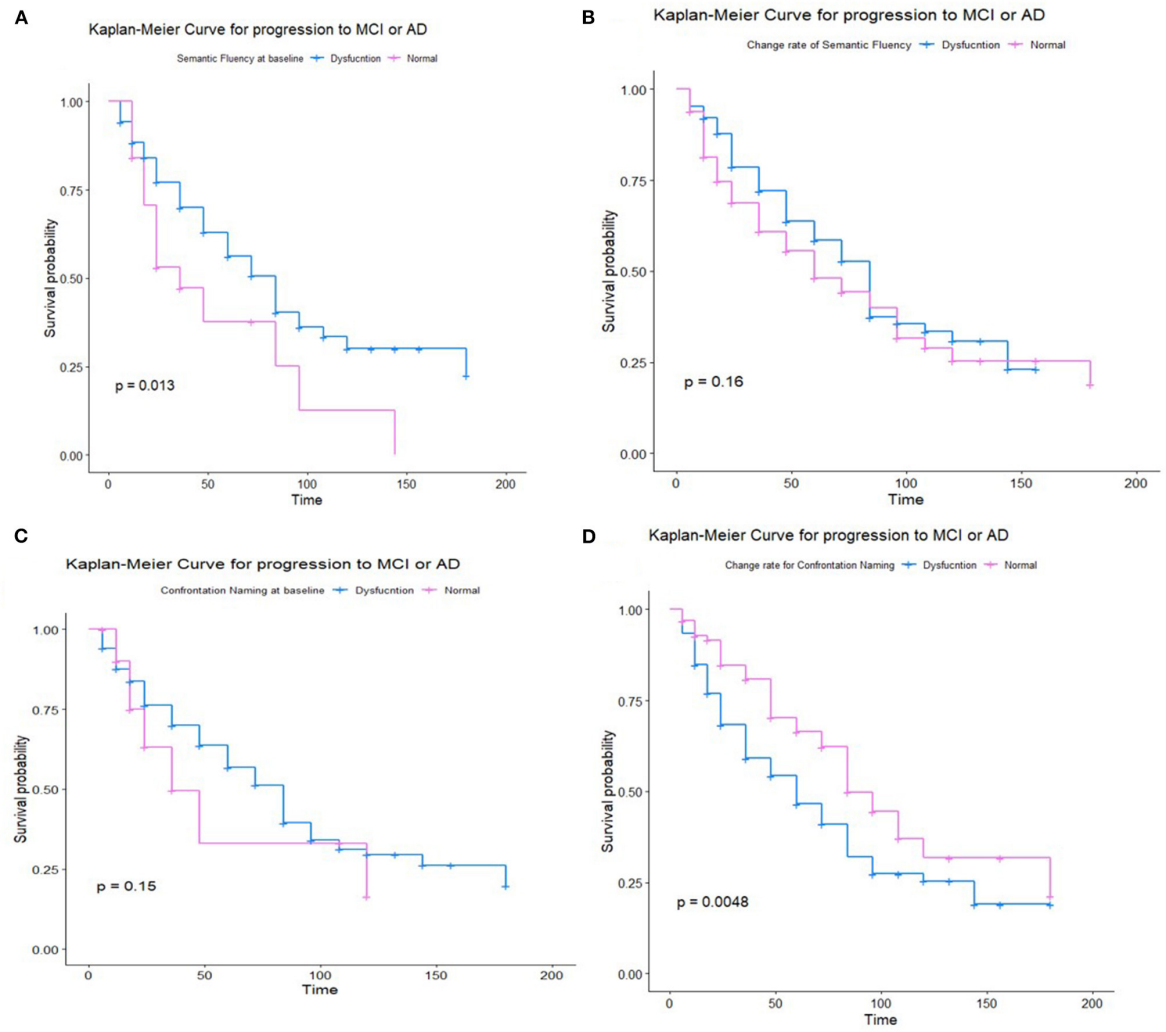
Kaplan–Meier survival analysis for non-demented participants for semantic fluency at the baseline **(A)**, the change rate of semantic fluency **(B)**, Kaplan–Meier survival analysis for non-demented participants for confrontation naming at baseline **(C)**, and the change rate of confrontation naming **(D)**.

The Kaplan–Meier survival analysis showed that the worse performance on semantic fluency was significantly associated with a shorter estimated time of progression to MCI or AD (*p* = 0.0013), while the performance on confrontation naming was not so significant (*p* = 0.15). The greater decline in confrontation naming was correlated with a higher risk of progression (*p* = 0.0048), while the decline in semantic fluency was not so significant (*p* = 0.16) (Figure 3). Subgroup analysis showed a greater decline in both semantic fluency and confrontation naming with higher progression to AD (Supplementary Figure S2).

### Language dysfunction among ATN profiles

**Figure 4:**
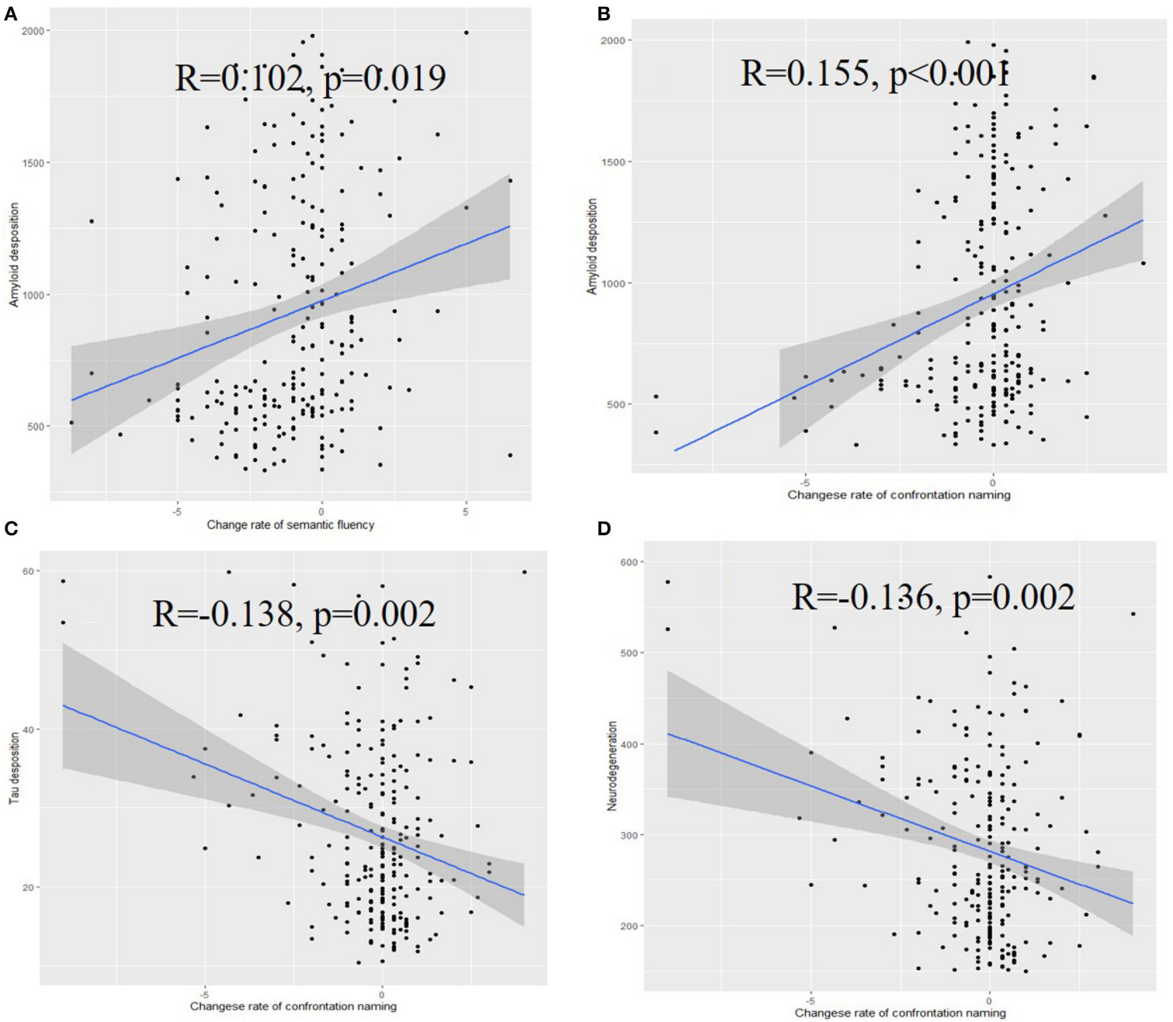
Associations of the rate of change of semantic fluency **(A)**, confrontation naming **(B)** with the amyloid deposition, associations of the rate of change of confrontation naming with tau deposition **(C)**, and neurodegeneration **(D)**.

These results suggest that rates of change in semantic fluency (*R* = 0.102, *p* = 0.019) and confrontation naming (*R* = 0.155, *p* < 0.001) were significantly associated with the baseline Aβ level. Notably, the Aβ-positive pathology was associated with significantly decreased levels of confrontation naming and semantic fluency. Significant associations were also noticed between the confrontation naming change rate and baseline tau level (*R* = −0.138, *p* = 0.002) or baseline neurodegeneration (*R* = −0.136, *p* = 0.002) (Figure 4), while the association between semantic fluency and tau level was non-significant (Supplementary Figure S1).

In multivariable analysis, the levels of Aβ (β = 0.171, *p* = 0.002), p-tau (β = −0.184, *p* = 0.003), and tau (β = −0.179, *p* = 0.003) were associated with the change rate of confrontation naming, while non-significant results were found for the change rate of semantic fluency (Supplementary Table 5).

**Figure 5:**
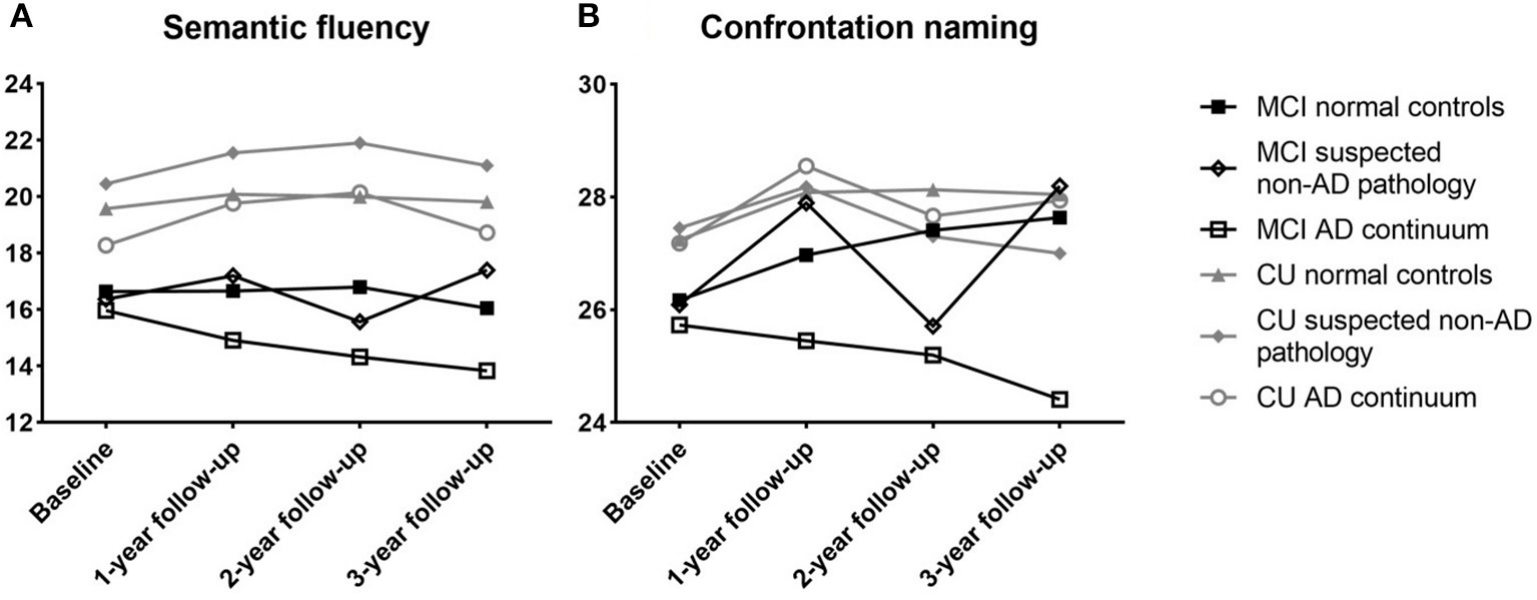
Performance of semantic fluency **(A)** and confrontation naming **(B)** for non-demented participants between different ATN profiles. CU, cognitive unimpaired; MCI, mild cognitive impairment.

Longitudinal analysis revealed a greater decline in confrontation naming for MCI subjects with different ATN profiles at 1-year (*F* (2,153) = 4.302, *p* = 0.015), 2-year (*F* (2,131) = 3.982, *p* = 0.021), and 3-year (*F* (2.84) = 5.156, *p* = 0.008) follow-ups. There was no significant difference between the semantic fluency at 1-year (*F* (2,164) = 0.317, *p* = 0.729), 2-year (*F* (2,153) = 2.257, *p* = 0.108), and 3-year (*F* (2.85) = 2.496, *p* = 0.089) follow-ups. Among the CU participants, worse but not significant performance differences were observed (Figure 5).

### Mediation analysis

The total, direct, and indirect associations of the change rate of confrontation naming with amyloid and tau deposition were assessed separately based on the schematic model shown in Figure 1. The proportion of association mediated between the change rate of confrontation naming and the probability of clinical progression mediated by Aβ42 and tau were 29% and 22%, respectively (Table 1), indicating that the greater decline in confrontation naming could be related to both amyloid degeneration and neurodegeneration. Although non-significant results were found for the change rate of semantic fluency through mediation analysis, subgroup analysis also found that the proportion for the clinical progression from MCI to AD was 43%, indicating amyloid deposition could modulate the decline in semantic fluency from MCI to AD (Supplementary Table 6).

**Table 1:** Adjusted direct and indirect associations of the change rate of confrontation naming with clinical progression via Aβ42, p-tau or t-tau.

**Measure**	**The change rate of confrontation naming**		**The change rate of semantic fluency**	
	β **(95%CI)**	***p***	β **(95%CI)**	***p***
**A**β**42**				
Total association	−0.03 (−0.07 to 0.00)	0.08	−0.04 (−0.08 to −0.02)	< 0.001^***^
Direct association	−0.02 (−0.06 to 0.01)	0.675	−0.04 (−0.04 to −0.01)	< 0.001^***^
Indirect association via Aβ42	−0.01 (−0.02 to 0.00)	0.04^*^	−0.01 ( −0.01 to 0.00)	0.2
Proportion mediated, %	29		6	
**P-tau**				
Total association	−0.03 (−0.08 to 0.01)	0.16	−0.04 (−0.07 to −0.02)	< 0.001^***^
Direct association	−0.02 (−0.08 to 0.02)	0.24	−0.04 (−0.06 to −0.01)	< 0.001^***^
Indirect association via p-tau	−0.01 (−0.02 to 0.00)	0.10	−0.01 ( −0.02 to 0.00)	0.2
Proportion mediated, %	22		6	
**Tau**				
Total association	−0.03 (−0.07 to 0.00)	0.08	−0.04 (−0.07 to −0.01)	< 0.001^***^
Direct association	−0.02 (−0.07 to 0.01)	0.30	−0.04 (−0.07 to −0.01)	< 0.001^***^
Indirect association via Tau	−0.01 (−0.02 to 0.00)	0.02^*^	−0.01 (−0.01 to 0.00)	0.14
Proportion mediated, %	27		7	

* indicates significance at *p* < 0.05.

*** indicates significance at *p* ≤ 0.001.

## Discussion

Our study confirmed that worse performance of language dysfunction was a significant risk factor for the clinical progression of cognitive impairments. Furthermore, the influence of semantic fluency and confrontation naming on the cognition and risk of pathological conversion to MCI or AD was partially mediated by amyloid pathology, while the confrontation naming itself could be mediated by tau pathology and neurodegeneration. All these findings thus supported the fact that the greater decline of semantic fluency might provide imperative information for early detection and intervention, while the cerebral amyloid deposition might mediate the effect of language dysfunction on cognitive impairment. The greater decline in confrontation naming could be related to both tau pathology and neurodegeneration.

Language dysfunction has been identified as an important indicator of preclinical AD detection (23). Our findings further confirmed the association between the change rates of semantic fluency and cognition and altered executive function before the onset of clinically significant cognitive dysfunctions, indicating that semantic fluency could be used as an imperative predictor for the disease progression rate. As a quick and relatively simple test, semantic fluency has been recommended as an accurate and efficient tool for screening early dementia symptoms (24, 25), in line with the worse performance for semantic fluency with a higher risk of progression. Recent studies have also confirmed the diagnostic accuracy of language assessment, semantic fluency as an example, for participants without dementia, even by automated detection (26, 27). Naming difficulties have also been observed in AD patients, attributing to the progressive degradation of semantic knowledge memory, which might be the reason underlying the non-significance of worse performance with the higher risk of progression (28). Semantic fluency tasks allow the identification of lexical–semantic impairment, deficits in semantic and working memory, as well as impaired executive function (29), while confrontation naming heavily relies on processing speed, learning, and memory (30). Our results highlighted that not only the baseline of semantic fluency and confrontation naming but also the change rates of semantic fluency and confrontation naming positively correlated with the decline of memory and executive function.

Although our longitudinal findings confirmed the greater decline in semantic fluency in the AD continuum, differences among ATN profiles were not significant. The mediation analysis showed that the influence of semantic fluency was partially mediated by the amyloid pathology from MCI to AD but not by tau pathology or neurodegeneration. Therefore, semantic fluency could be a sensitive biomarker for identifying early AD symptoms and predicting the risk of pathological conversion of MCI into AD (31). Previous studies indicate that there have been significant differences in terms of semantic fluency between MCI subjects with amyloidosis and AD patients (32, 33). However, it is reported that semantic fluency lack specificity and cannot differentiate between AD and other forms of dementia, including Parkinson's disease (34, 35). Moreover, a steeper decline in semantic fluency could be observed between Aβ status approximately 2.5 years before neuroimaging by Aβ-PET in non-demented participants (36). Similar to our results, the meta-analysis also confirmed that semantic fluency is associated with amyloid burden, particularly for those studies which did not select individuals with subjective impairment (37). One possible reason behind the weak association between amyloid accumulation and semantic fluency impairment at the earliest stage might be the sigmoid curve of amyloid accumulation (38). It might be possible that semantic fluency might be more sensitive to amyloid pathophysiology in a diffuse manner, which would be obvious at a late stage after abundant amyloid accumulation, and more studies are needed to reveal the possible mechanism underlying the dysfunction of semantic fluency and amyloid accumulation. Taken together, these findings suggest that the semantic fluency decline could be regarded as an early sign of AD spectrum (39); however, there might be some overlapping symptoms during the advancement of disease courses for different types of dementia

In terms of confrontation naming, our longitudinal findings confirmed the greater decline among the participants with different ATN profiles. The mediation analysis also revealed that the influence of confrontation naming was correlated with amyloidosis, tau pathology, and neurodegeneration. Our results exhibited that confrontation naming was partially mediated by both amyloidosis and tau pathology. Previous studies also reported statistically significant associations in terms of the decline in confrontation naming only when both amyloidosis and hippocampal atrophy were evident (40). Variations in confrontation naming are primarily shared between the tau pathology and brain atrophy (51%) for primary progressive aphasia with underlying AD pathology, especially in the left anterior temporal lobe (32), indicating a more specific dysfunction in the language network compared with semantic fluency. The hypometabolism of the left temporal lobe, the temporopolar cortex in particular, demonstrated severe confrontation naming impairment (41), indicating that the temporopolar cortex might act as an important semantic hub in the language network. Not only the indirect measure of tau PET (42) but also three-dimensional quantitative maps of neurofibrillary tangle burden confirmed the roles of tau burden in the temporal lobe, especially in the temporopolar cortex (43, 44). Collectively, the decline in confrontation naming could be a potential biomarker for both tau burden and amyloidosis, in particular in the temporopolar cortex. The main strength of our article is to include longitudinal assessments of language impairment, clinical progression, pathological mechanisms, and causal mediation analyses. Our analysis suggested that participants with a faster decline of language impairment might suggest clinical progression, while the rehabilitation of language impairment might delay or slow the clinical progression. In participants with worse confrontation naming, amyloid and tau deposition may act together to contribute to clinical progression, while amyloid deposition may contribute to the progression from MCI to AD in participants with a decline of semantic fluency. Confrontation naming is related to more specific dysfunction marked by tau and atrophy, while semantic fluency might be more sensitive to amyloid pathophysiology, especially from MCI to AD.

There are also certain limitations in this study. First, the BNT and AFT were chosen to evaluate the severity of language dysfunction in the present study, which was only a part of the clinical features of language dysfunction, and thus prevented us from analyzing the acoustic features of the participants. Moreover, the effect of other potential confounding factors (e.g., other genetic factors and vascular risk factors) was not included in our analyses, which might also be the reason underlying the relatively low correlation coefficient. Linear regression was further conducted to confirm the roles of language dysfunction in global cognition, executive function, and ATN biomarkers. Similar results confirmed the stability of correlation analysis. Therefore, further large-scale community-based longitudinal studies are warranted to validate these associations, especially when both lexical and acoustic features of the participants are considered.

## Data availability statement

The data analyzed in this study was obtained from Alzheimer's Disease Neuroimaging Initiative (ADNI), the following licenses/restrictions apply: Access to the datasets is contingent on adherence to the ADNI Data Use Agreement and publications' policies and the submission of an online application form. The application must include the investigator's institutional affiliation and the proposed uses of the ADNI data. ADNI data may not be used for commercial products or redistributed in any way. Requests to access these datasets should be directed to ADNI, https://adni.loni.usc.edu/data-samples/access-data/.

## Ethics statement

The studies involving human participants were reviewed and approved by ADNI, and were conducted in accordance with the Good Clinical Practice guidelines, the Declaration of Helsinki, and US 21 CFR: Part 50 (Protection of Human Subjects) and Part 56 (Institutional Review Boards). The ADNI study was conducted in compliance with HIPAA regulations. CX was granted administrative permissions to access the anonymized ADNI data in September, 2022. Ethical approval for data collection was obtained by each ADNI participating institution's review board. The review boards of each institution were: Oregon Health and Science University; University of Southern California; University of California—San Diego; University of Michigan; Mayo Clinic, Rochester; Baylor College of Medicine; Columbia University Medical Center; Washington University, St. Louis; University of Alabama at Birmingham; Mount Sinai School of Medicine; Rush University Medical Center; Wien Center; Johns Hopkins University; New York University; Duke University Medical Center; University of Pennsylvania; University of Kentucky; University of Pittsburgh; University of Rochester Medical Center; University of California, Irvine; University of Texas Southwestern Medical School; Emory University; University of Kansas, Medical Center; University of California, Los Angeles; Mayo Clinic, Jacksonville; Indiana University; Yale University School of Medicine; McGill University, Montreal Jewish General Hospital; Sunnybrook Health Sciences, Ontario; U.B.C.Clinic for AD & Related Disorders; Cognitive Neurology—St. Joseph's, Ontario; Cleveland Clinic Lou Ruvo Center for Brain Health; Northwestern University; Premiere Research Inst (Palm Beach Neurology); Georgetown University Medical Center; Brigham and Women's Hospital; Stanford University; Banner Sun Health Research Institute; Boston University; Howard University; Case Western Reserve University; University of California, Davis-Sacramento; Neurological Care of CNY; Parkwood Hospital; University of Wisconsin; University of California, Irvine-BIC; Banner Alzheimer's Institute; Dent Neurologic Institute; Ohio State University; Albany Medical College; Hartford Hospital, Olin Neuropsychiatry Research Center; Dartmouth-Hitchcock Medical Center; Wake Forest University Health Sciences; Rhode Island Hospital; Butler Hospital; UC San Francisco; Medical University South Carolina; St. Joseph's Health Care Nathan Kline Institute; University of Iowa College of Medicine; Cornell University; and University of South Florida: USF Health Byrd Alzheimer's Institute. All study participants or authorized representatives provided written informed consent to participate in this study.

## Author contributions

CX and WA took responsibility for the calculation and draft the article. YZ took responsibility for the design of the study, full-text evaluation, and guidance. All authors read and approved the final manuscript.

## Group members of Alzheimer's Disease Neuroimaging Initiative

Data used in preparation for this article were obtained from the Alzheimer's Disease Neuroimaging Initiative (ADNI) database (adni.loni.usc.edu). As such, the investigators within the ADNI contributed to the design and implementation of ADNI and/or provided data but did not participate in the analysis or writing of this report. A complete listing of ADNI investigators can be found in the Supplementary material.
