# Supplementary material for: Language dysfunction correlates with cognitive impairments in older adults without dementia mediated by amyloid pathology
Source: Front Neurol. 2023 May 17;14:1051382. doi: 10.3389/fneur.2023.1051382 (PMC10230042; doi:10.3389/fneur.2023.1051382)
Supplement: Supplementary file 1 [file Table_1.docx]

Supplementary table 1 Cross-sectional associations of semantic fluency with demographic characteristics, neuropsychological assessment, and CSF biomarkers among non-demented participants

|  | CU- | | MCI- | | CU+ | | MCI+ | | Total | |
| --- | --- | --- | --- | --- | --- | --- | --- | --- | --- | --- |
|  |  |  |  |  | (preclinical AD) | | (prodromal AD) | |  |  |
|  | β | P | β | P | β | P | β | P | β | P |
| Demographics | | | | | | | | | | |
| Age of visit | -0.146 | 0.232 | -0.092 | 0.510 | -0.209 | 0.236 | -0.176 | 0.059 | -0.072 | 0.098 |
| Gender | -0.077 | 0.452 | 0.121 | 0.310 | -0.011 | 0.944 | -0.050 | 0.525 | -0.001 | 0.981 |
| Education of years | 0.423 | ＜0.001* | -0.052 | 0.708 | 0.269 | 0.124 | 0.075 | 0.426 | 0.115 | 0.009* |
| APOE status | 0.195 | 0.058 | -0.094 | 0.426 | -0.044 | 0.767 | 0.039 | 0.605 | -0.053 | 0.386 |
| Global cognition | | | | | | | | | | |
| MMSE | 0.171 | 0.160 | 0.120 | 0.259 | 0.076 | 0.671 | 0.221 | 0.017* | 0.202 | ＜0.001* |
| ADAS-Cog | -0.226 | 0.062 | -0.177 | 0.074 | -0.086 | 0.628 | -0.243 | 0.009* | -0.238 | ＜0.001* |
| Memory | | | | | | | | | | |
| RAVLT immediate recall | 0.182 | 0.134 | 0.133 | 0.179 | 0.130 | 0.315 | 0.231 | 0.013* | 0.279 | ＜0.001* |
| RAVLT learning | 0.102 | 0.406 | 0.122 | 0.236 | 0.167 | 0.212 | 0.102 | 0.277 | 0.168 | ＜0.001* |
| RAVLT delayed recall | 0.107 | 0.383 | 0.224 | 0.030* | 0.220 | 0.095 | 0.079 | 0.403 | 0.270 | ＜0.001* |
| RAVLT delayed recognition | 0.083 | 0.498 | 0.096 | 0.350 | 0.131 | 0.339 | 0.077 | 0.412 | 0.214 | ＜0.001* |
| Attention/Executive function | | | | | | | | | | |
| TMT Part A | -0.253 | 0.036* | -0.023 | 0.816 | -0.268 | 0.040 | -0.197 | 0.036 | -0.197 | ＜0.001* |
| TMT Part B | -0.313 | 0.009* | -0.027 | 0.781 | -0.158 | 0.218 | -0.363 | ＜0.001* | -0.238 | ＜0.001* |
| Processing speed | | | | | | | | | | |
| Digit span forward | 0.230 | 0.058 | -0.090 | 0.403 | -0.009 | 0.949 | 0.044 | 0.642 | 0.082 | 0.081 |
| Digit span backward | 0.277 | 0.060 | 0.121 | 0.256 | 0.165 | 0.233 | 0.079 | 0.403 | 0.119 | 0.010* |
| Visuospatial | | | | | | | | | | |
| CDT copy | 0.227 | 0.030 | -0.010 | 0.941 | -0.010 | 0.941 | 0.112 | 0.128 | 0.125 | 0.039* |
| CDT command | 0.050 | 0.634 | -0.207 | 0.422 | -0.207 | 0.422 | -0.207 | 0.202 | 0.008* | 0.016* |
| Neuropsychology | | | | | | | | | | |
| NPI | -0.131 | 0.283 | -0.125 | 0.367 | -0.187 | 0.167 | 0.129 | 0.170 | 0.123 | 0.011* |
| GDS | 0.030 | 0.806 | -0.175 | 0.204 | 0.019 | 0.888 | 0.156 | 0. 096 | 0.180 | 0.003* |
| Functional activity | | | | | | | | | | |
| FAQ | 0.067 | 0.582 | -0.046 | 0.741 | -0.115 | 0.448 | -0.098 | 0.298 | -0.161 | 0.001* |

Abbreviations: CU-, cognitively unimpaired with negative Aβ; MCI, mild cognitive impairment; RAVLT, Rey Auditory Semantic Learning Test; CDT, Clock Drawing Test; FAQ, Functional Activity Questionaire; NPI, Neuropsychiatric Inventory Questionnaire; GDS, Geriatric Depression Scale and Neuropsychiatric Inventory Questionnaire; TMT part A and B, Trail Making Test Part A and B; MMSE, Mini-Mental State Examination; ADAS-Cog, AD Assessment Schedule-Cognition
