# Supplementary material for: Language dysfunction correlates with cognitive impairments in older adults without dementia mediated by amyloid pathology
Source: Front Neurol. 2023 May 17;14:1051382. doi: 10.3389/fneur.2023.1051382 (PMC10230042; doi:10.3389/fneur.2023.1051382)
Supplement: Supplementary file 2 [file Table_2.docx]

Supplementary table2. Cross-sectional associations of confrontation naming with demographic characteristics, neuropsychological assessment, and CSF biomarkers among non-demented participants

|  | CU- | | MCI- | | CU+ | | MCI+ | | Total | |
| --- | --- | --- | --- | --- | --- | --- | --- | --- | --- | --- |
|  |  |  |  |  | (preclinical AD) | | (prodromal AD) | |  |  |
|  | β | P | β | P | β | P | β | P | β | P |
| Demographics | | | | | | | | | | |
| Age of visit | 0.152 | 0.213 | -0.153 | 0.269 | -0.056 | 0.668 | -0.207 | 0.026 | -0.082 | 0.177 |
| Gender | -0.220 | 0.039 | 0.121 | 0.310 | -0.011 | 0.944 | -0.026 | 0.746 | -0.001 | 0.981 |
| Education of years | 0.394 | ＜0.001* | -0.019 | 0.890 | -0.142 | 0.293 | 0.112 | 0.234 | 0.089 | 0.145 |
| APOE status | -0.031 | 0.767 | -0.094 | 0.426 | -0.044 | 0.010 | 0.197 | 0.010 | -0.053 | 0.386 |
| Global cognition |  |  |  |  |  |  |  |  |  |  |
| MMSE | 0.228 | 0.059 | 0.049 | 0.557 | 0.157 | 0.285 | 0.295 | 0.001* | 0.296 | ＜0.001* |
| ADAS-Cog | -0.330 | 0.006* | -0.265 | 0.053 | -0.356 | ＜0.001* | -0.356 | ＜0.001* | -0.266 | ＜0.001* |
| Memory | | | | | | | | | | |
| RAVLT immediate recall | 0.182 | 0.134 | 0.180 | 0.192 | 0.254 | 0.147 | 0.217 | 0.020* | 0.292 | ＜0.001* |
| RAVLT learning | 0.102 | 0.406 | 0.139 | 0.317 | 0.091 | 0.610 | 0.105 | 0.266 | 0.200 | ＜0.001* |
| RAVLT delayed recall | 0.107 | 0.383 | 0.280 | 0.040* | 0.301 | 0.084 | 0.053 | 0.575 | 0.253 | ＜0.001* |
| RAVLT delayed recognition | 0.083 | 0.498 | 0.164 | 0.236 | 0.611 | ＜0.001* | 0.035 | 0.710 | 0.232 | ＜0.001* |
| Attention/Executive function | | | | | | | | | | |
| TMT Part A | -0.253 | 0.036* | -0.392 | 0.003 | -0.435 | 0.010* | -0.256 | 0.006 | -0.315 | ＜0.001* |
| TMT Part B | -0.313 | 0.009* | -0.496 | ＜0.001* | -0.471 | 0.005* | -0.487 | ＜0.001* | -0.479 | ＜0.001* |
| Processing speed | | | | | | | | | | |
| Digit span forward | 0.230 | 0.058 | -0.096 | 0.491 | 0.002 | 0.992 | 0.067 | 0.478 | 0.073 | 0.229 |
| Digit span backward | 0.240 | 0.047* | 0.193 | 0.162 | 0.066 | 0.711 | 0.105 | 0.263 | 0.175 | 0.004* |
| Visuospatial | | | | | | | | | | |
| CDT copy | 0.227 | 0.030* | 0.337 | 0.014 | 0.583 | 0.112 | 0.112 | 0.128 | 0.279 | ＜0.001* |
| CDT command | 0.050 | 0.634 | 0.112 | 0.422 | 0.090 | 0.545 | -0.207 | 0.202 | 0.152 | 0.003 |
| Neuropsychology | | | | | | | | | | |
| NPI | 0.114 | 0.412 | 0.114 | 0.412 | -0.017 | 0.925 | 0.190 | 0.042* | 0.065 | 0.179 |
| GDS | -0.024 | 0.866 | -0.024 | 0.866 | -0.343 | 0.047* | -0.008 | 0.935 | 0.146 | 0.002* |
| Functional activity | | | | | | | | | | |
| FAQ | -0.046 | 0.741 | -0.046 | 0.741 | -0.105 | 0.556 | -0.063 | 0.501 | -0.161 | 0.001* |

Abbreviations: CU-, cognitively unimpaired with negative Aβ; MCI, mild cognitive impairment; RAVLT, Rey Auditory Semantic Learning Test; CDT, Clock Drawing Test; FAQ, Functional Activity Questionaire; NPI, Neuropsychiatric Inventory Questionnaire; GDS, Geriatric Depression Scale and Neuropsychiatric Inventory Questionnaire; TMT part A and B, Trail Making Test Part A and B; MMSE, Mini-Mental State Examination; ADAS-Cog, AD Assessment Schedule-Cognition
