# Supplementary material for: Language dysfunction correlates with cognitive impairments in older adults without dementia mediated by amyloid pathology
Source: Front Neurol. 2023 May 17;14:1051382. doi: 10.3389/fneur.2023.1051382 (PMC10230042; doi:10.3389/fneur.2023.1051382)
Supplement: Supplementary file 3 [file Table_3.docx]

|  | Global cognition | | | Executive function | | |
| --- | --- | --- | --- | --- | --- | --- |
|  | β | t | *p* | β | t | *p* |
| Confrontation naming |  |  |  |  |  |  |
| Sex | -0.211 | -3.783 | ＜0.001*** | -0.116 | -2.027 | 0.044* |
| Age | -0.054 | -0.975 | 0.331 | -0.001 | 0.160 | 0.992 |
| Education | -0.077 | -1.384 | 0.167 | -0.015 | -0.565 | 0.797 |
| APOE | 0.283 | 5.195 | ＜0.001*** | 0.030 | 0.447 | 0.592 |
| Confrontation naming | -0.361 | -6.651 | ＜0.001*** | -0.466 | -8.792 | ＜0.001*** |
| Semantic fluency |  |  |  |  |  |  |
| Sex | -0.205 | -3.578 | ＜0.001*** | -0.113 | -1.836 | 0.068 |
| Age | -0.063 | -1.121 | 0.263 | -0.004 | -0.067 | 0.947 |
| Education | -0.063 | -1.090 | 0.277 | -0.011 | -0.170 | 0.865 |
| APOE | 0.254 | 4.522 | ＜0.001*** | 0.002 | 0.025 | 0.980 |
| Semantic fluency | -0.316 | -5.567 | ＜0.001*** | -0.321 | -5.267 | ＜0.001*** |

Supplementary Table S3

Association between global cognition and executive functions with bsemantic fluency and confrontation naming among participants without dementia

* indicates significance at p < 0.05. ** indicates significance at p ≤ 0.01. *** indicates significance at p≤ 0.001
