# Supplementary material for: Language dysfunction correlates with cognitive impairments in older adults without dementia mediated by amyloid pathology
Source: Front Neurol. 2023 May 17;14:1051382. doi: 10.3389/fneur.2023.1051382 (PMC10230042; doi:10.3389/fneur.2023.1051382)
Supplement: Supplementary file 4 [file Table_4.docx]

|  | Global cognition | | | Executive function | | |
| --- | --- | --- | --- | --- | --- | --- |
|  | β | t | *p* | β | t | *p* |
| The change rate of confrontation naming |  |  |  |  |  |  |
| Sex | -0.207 | -3.386 | 0.001** | -0.145 | -2.243 | 0.026 |
| Age | -0.007 | -0.123 | 0.903 | 0.068 | 1.073 | 0.284 |
| Education | -0.114 | -1.893 | 0.060 | -0.086 | -1.350 | 0.178 |
| APOE | 0.287 | 4.814 | ＜0.001*** | 0.028 | 0.448 | 0.655 |
| Change rate of confrontation naming | -0.174 | -2.953 | 0.003** | -0.213 | -3.421 | ＜0.001*** |
| The change rate of semantic fluency |  |  |  |  |  |  |
| Sex | -0.215 | -3.639 | ＜0.001*** | -0.124 | -2.010 | 0.045* |
| Age | -0.034 | -0.592 | 0.554 | 0.022 | 0.357 | 0.721 |
| Education | -0.129 | -2.197 | 0.029 | -0.083 | -1.354 | 0.177 |
| APOE | 0.253 | 4.315 | ＜0.001*** | -0.017 | -0.281 | 0.779 |
| Semantic fluency | -0.203 | -3.488 | 0.001** | -0.302 | -4.967 | ＜0.001*** |

Supplementary Table S4

Association between global cognition and executive functions with the change rate of semantic fluency and confrontation naming among participants without dementia

* indicates significance at p < 0.05. ** indicates significance at p ≤ 0.01. *** indicates significance at p≤ 0.001
