# Supplementary material for: Language dysfunction correlates with cognitive impairments in older adults without dementia mediated by amyloid pathology
Source: Front Neurol. 2023 May 17;14:1051382. doi: 10.3389/fneur.2023.1051382 (PMC10230042; doi:10.3389/fneur.2023.1051382)
Supplement: Supplementary file 5 [file Table_5.docx]

| Variable | Aβ42 | | | Tau | | | P-tau | | |
| --- | --- | --- | --- | --- | --- | --- | --- | --- | --- |
|  | β | t | *p* | β | t | *p* | β | t | *p* |
| The change rate of confrontation naming | | | | | | | | | |
| Sex | .077 | 1.360 | .175 | .001 | .013 | .990 | -.002 | -.029 | .977 |
| Age | .023 | .420 | .675 | .087 | 1.449 | .149 | .060 | 1.002 | .317 |
| Education | -.005 | -.096 | .924 | -.083 | -1.361 | .175 | -.067 | -1.109 | .268 |
| APOE | -.430 | -7.676 | .000 | .276 | 4.539 | .000 | .304 | 5.015 | .000 |
| Change rate of confrontation naming | .171 | 3.074 | .002 | -.184 | -3.047 | .003 | -.179 | -2.994 | .003 |
| The change rate of semantic fluency | | | | | | | | | |
| Sex | .058 | .985 | .326 | .029 | .454 | .650 | .031 | .498 | .619 |
| Age | .004 | .073 | .942 | .099 | 1.577 | .116 | .073 | 1.172 | .242 |
| Education | -.016 | -.271 | .787 | -.062 | -.980 | .328 | -.043 | -.684 | .495 |
| APOE | -.466 | -8.154 | .000 | .311 | 4.997 | .000 | .343 | 5.571 | .000 |
| Semantic fluency | .079 | 1.400 | .163 | -.087 | -1.416 | .158 | -.088 | -1.444 | .150 |

Supplementary Table S5

Association between biological data with the change rate of semantic fluency and confrontation naming among participants without dementia

* indicates significance at p < 0.05. ** indicates significance at p ≤ 0.01. *** indicates significance at p≤ 0.001
