# Supplementary material for: Language dysfunction correlates with cognitive impairments in older adults without dementia mediated by amyloid pathology
Source: Front Neurol. 2023 May 17;14:1051382. doi: 10.3389/fneur.2023.1051382 (PMC10230042; doi:10.3389/fneur.2023.1051382)
Supplement: Supplementary file 6 [file Table_6.docx]

Supplementary Table 6

Adjusted direct and indirect associations of the change rate of confrontation naming with clinical progression via Aβ42, p-tau or t-tau among MCI participants

| Measure | The change rate of confrontation naming | | The change rate of semantic fluency | |
| --- | --- | --- | --- | --- |
|  | β(95%CI) | *p* | β(95%CI) | *p* |
| Aβ42 |  | |  |  |
| Total association | -0.03(-0.09 to 0.00) | 0.06 | -0.04(-0.06 to 0.00) | 0.04* |
| Direct association | -0.01(-0.07 to 0.03) | 0.54 | -0.02(-0.03 to 0.00) | 0.04* |
| Indirect association via Aβ42 | -0.01( -0.07 to -0.01) | ＜0.001*** | -0.01( -0.01 to 0.00) | ＜0.001*** |
| Proportion mediated, % | 70 |  | 43 |  |
| P-tau |  |  |  |  |
| Total association | -0.03(-0.08 to 0.01) | 0.18 | -0.04(-0.07 to 0.00) | 0.06 |
| Direct association | -0.03(-0.08 to 0.01) | 0.22 | -0.04(-0.07 to 0.01) | 0.10 |
| Indirect association via p-tau | -0.01(-0.02 to 0.00) | 0.46 | -0.01( -0.01 to 0.00) | 0.34 |
| Proportion mediated, % | 9 |  | 6 |  |
| Tau |  |  |  |  |
| Total association | -0.03(-0.09 to 0.01) | 0.16 | -0.04(-0.07 to 0.00) | 0.06 |
| Direct association | -0.02(-0.09 to 0.02) | 0.18 | -0.03(-0.07 to 0.00) | 0.06 |
| Indirect association via Tau | -0.01(-0.02 to 0.00) | 0.30 | -0.01(-0.01 to 0.00) | 0.38 |
| Proportion mediated, % | 16 |  | 7 |  |

* indicates significance at p < 0.05. ** indicates significance at p ≤ 0.01. *** indicates significance at p≤ 0.001
